# A Structural Optimization Framework for Biodegradable Magnesium Interference Screws

**DOI:** 10.3390/biomimetics10040210

**Published:** 2025-03-28

**Authors:** Zhenquan Shen, Xiaochen Zhou, Ming Zhao, Yafei Li

**Affiliations:** 1 Faculty of Artificial Intelligence in Education, Central China Normal University, Wuhan 430079, China; 2 School of Materials Science and Engineering, Peking University, Beijing 100871, China; 3 Schlumberger Technology Corporation, Houston, TX 77054, USA; 4 School of Materials Science and Engineering, Zhengzhou University, Zhengzhou 450001, China

**Keywords:** biodegradation, magnesium alloys, interference screw, continuum damage mechanics, finite element method, structural optimization

## Abstract

Biodegradable magnesium alloys have garnered increasing attention in recent years, with magnesium alloy–based biomedical devices being clinically used. Unlike biologically inert metallic materials, magnesium-based medical devices degrade during service, resulting in a mechanical structure that evolves over time. However, there are currently few computer-aided engineering methods specifically tailored for magnesium-based medical devices. This paper introduces a structural optimization framework for Mg-1Ca interference screws, accounting for degradation using a continuum damage model (CDM). The Optimal Latin Hypercube Sampling (OLHS) technique was employed to sample within the design space. Pull-out strengths were used as the optimization objective, which were calculated through finite element analysis (FEA). Both Response Surface Methodology (RSM) and Kriging models were employed as surrogate models and optimized using the Sequential Quadratic Programming (SQP) algorithm. The results from the Kriging model were validated through FEA, and were found to be acceptable. The relationships between the design parameters, the rationale behind the methodology, and its limitations are discussed. Finally, a final design is proposed along with recommendations for interference screw design.

## 1. Introduction

Magnesium (Mg) and its alloys hold significant potential as next-generation biomedical implants due to their favorable mechanical properties, inherent biocompatibility, and, most importantly, biodegradability [1,2,3,4,5]. Unlike traditional permanent implants, Mg-based implants gradually degrade under physiological conditions while bone and tissue repair occurs. As an essential elements in the human body, the degradation products of Mg, primarily Mg ions, can be metabolized [6,7,8,9], making the degradation process biologically safe. This property eliminates the need for a secondary surgery to remove the implant after healing, thereby reducing both the physical pain and financial burdens associated with surgical complications.

Mg-based implants are primarily utilized in two key medical fields: orthopedics and cardiovascular medicine [10]. In cardiovascular interventions, late stent thrombosis remains a major concern due to the permanent presence of traditional stents. Additionally, permanent stents can impair vasomotion within the stented segment and negatively affect endothelial responses in adjacent vessel segments [11]. To address these challenges, Mg-based biodegradable stents have been developed. However, since Mg degrades in vivo, ensuring adequate radial force and fatigue endurance for a required period before vessel remodeling is crucial. Consequently, the structural design of Mg-based stents must differ from that of conventional permanent stents to achieve optimal performance. Several studies have explored the geometric design of Mg-based stents [12,13,14]. Grogan et al. optimized stent structures using a global optimization algorithm combined with an Arbitrary Lagrangian–Eulerian corrosion model to simulate stent degradation [12]. Chen et al. employed a global response surface method to control stent shape evolution [13], while Wu et al. utilized an adaptive response surface-based optimization algorithm [14].

In the realm of orthopedics, biomaterials find extensive application, serving as substitutes for bone tissue, devices for bone fixation, and systems for stabilizing fractures, alongside their use in repairing ligaments and tendons, and in the procedure of total hip replacement [15]. Currently, metal implants such as stainless steel, titanium alloys, and cobalt-chromium alloys are commonly used as permanent orthopedic devices. However, these materials may cause stress shielding, potentially leading to re-fracture and compromising the bio-efficacy of implants, particularly in osteoporotic patients [16,17]. Additionally, the long-term presence of permanent implants may introduce adverse effects after fracture healing [18]. While polymer-based implants have been developed to address some of these issues, they often suffer from inadequate mechanical strength and adverse tissue responses. Insufficient mechanical strength may lead to implant failure, while the degradation by-products of certain polymers can induce long-term inflammatory responses in surrounding tissues [19].

In contrast, Mg exhibits mechanical properties similar to cortical bone, helping to mitigate stress shielding and improve healing outcomes. Moreover, Mg ions can promote new bone formation, further enhancing fracture healing [20]. Among orthopedic implants, screws are among the most widely used. Enhancing the mechanical performance of Mg-based interference screws is as crucial as optimizing the design of cardiovascular stents. Unlike traditional metal interference screws, Mg-based screws gradually degrade in vivo, making their fixation dependent on the degradation process. Notably, the threaded portions of Mg-based screws, which bear mechanical loads at the screw-tissue interface, tend to degrade preferentially due to stress corrosion [21,22]. Therefore, the structural design of Mg-based screws must differ from that of bio-inert metallic screws, particularly in the threaded regions.

However, unlike stents, limited research has been conducted on the structural optimization of Mg-based screws. Optimizing their design is crucial not only for maximizing therapeutic efficacy, but also for facilitating their commercial viability. Computational simulations offer an effective approach for achieving optimal structural designs, allowing for the implementation of various optimization strategies. The challenge with Mg-based implants lies in integrating biodegradation into the optimization process. Since the performance of interference screws depends on both corrosion behavior and mechanical integrity [23,24], optimization strategies must incorporate corrosion models to simulate screw degradation.

Several corrosion models have been proposed to address this challenge. Gastaldi et al. [25] and Wu et al. [26] developed continuum damage models (CDM) within finite element frameworks to analyze uniform and stress-mediated corrosion in coronary stents. Grogan et al. extended this approach by introducing a pitting corrosion model [27], while Shen et al. further enhanced it by incorporating a corrosion-fatigue model [6]. Galvin et al. [28] developed a strain-mediated corrosion model. These corrosion models can be utilized to optimize Mg-based screw structures when combined with appropriate optimization strategies.

The pull-out strength of a screw is a key performance indicator and should be the primary objective of design optimization. In this study, we optimized the relationship between the geometric parameters and the pull-out strength of Mg-1Ca interference screws, both in their original state and after typical degradation. A CDM-based uniform corrosion model was integrated with a sequential quadratic programming (SQP) optimization strategy to refine the geometric design of Mg-1Ca interference screws. Both the response surface model (RSM) and Kriging model were used as surrogate models to establish the relationship between geometric parameters and pull-out strength. To the best of our knowledge, this is the first study to optimize the design of Mg-based interference screws while explicitly considering biodegradation.

## 2. Method

### 2.1. Design Parameters

**Figure 1:**
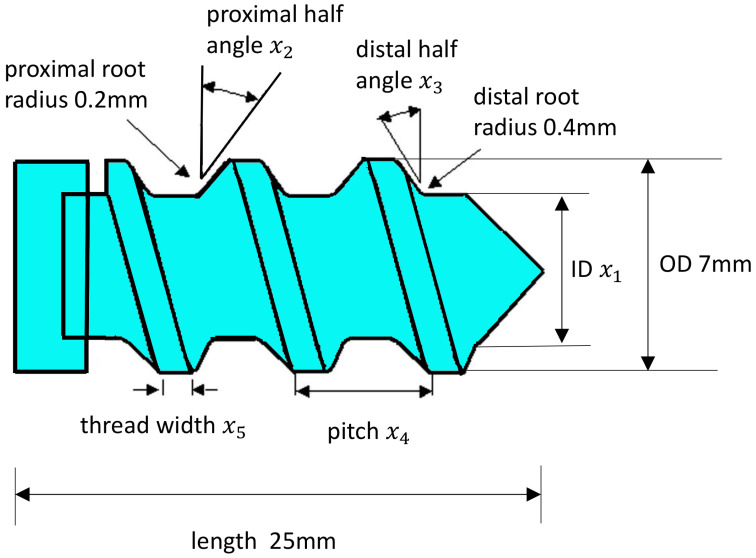
Schematic diagram of an interference screw. The head is on the left (proximal side of the screw).

The geometry of a typical interference screw is illustrated in the schematic diagram in Figure 1. In this study, five key design parameters were selected: inner diameter ($x_{1}$), proximal half-angle ($x_{2}$), distal half-angle ($x_{3}$), pitch ($x_{4}$), and thread width ($x_{5}$). Based on relevant commercial products, industrial standards, and manufacturing constraints, the bounds for these design parameters are defined in Equation (1), collectively establishing the design space for optimization.

(1) $$\left\{\begin{array}{c} 3.5\leq x_{1}\leq 5.0\;\mathrm{mm}\\ 15\leq x_{2}\leq 20{}^{\circ}\\ 30\leq x_{3}\leq 35{}^{\circ}\\ 2.2\leq x_{4}\leq 2.9\;\mathrm{mm}\\ 0.3\leq x_{5}\leq 0.6\;\mathrm{mm} \end{array}\right.$$

**Figure 2:**
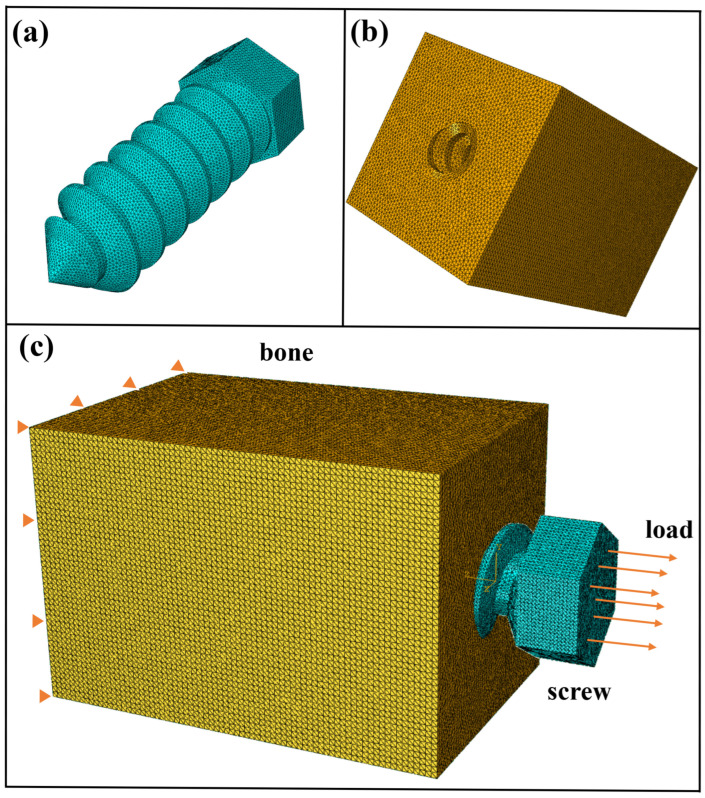
(**a**) Three-dimensional diagram of a screw; (**b**) three-dimensional diagram of a bone. (**c**) The screw is pulled in a rightward direction to measure pull-out strength (The triangle signs indicate that the displacement boundary condition is set to 0).

Parameters not included in the optimization study were kept constant based on data from available commercial products [29], including an outer diameter of 7 mm, a length of 25 mm, a proximal root radius of 0.2 mm, and a distal root radius of 0.4 mm. Figure 2a presents a three-dimensional diagram of the screw. Optimal Latin Hypercube Sampling (OLHS) is an efficient space-sampling technique that ensures comprehensive coverage of the design space [30,31]. In this study, OLHS was employed to generate sample points. The points listed in Table 1 were randomly generated within the design space, and their distribution was optimized by maximizing the distance between them, thereby representing the entire design space with the minimal number of sample points.

### 2.2. Corrosion Model and Model Calibration

A corrosion model based on the Continuum Damage Mechanics (CDM) model developed by Gastaldi et al. was adopted as a reference [25]. The core concept of CDM is to describe corrosion through a damage factor D, which is a function of spatial coordinates and time. Under the assumption of isotropy, D is a scalar field that reflects the material properties. Specifically, D=0 indicates an undamaged material with no corrosion, while D=1 represents a completely damaged material corresponding to 100% corrosion. The corrosion damage was modeled using the pitting corrosion model developed by Grogan et al. [27]. The damage evolution law is given by

(2) $$\frac{dD_{C}}{dt}=\frac{\delta_{U}}{L_{e}}\lambda_{e}K_{U}$$

where t signifies time and $D_{C}$ acts as the corrosion damage factor. $\delta_{U}$ defines a key dimension of the uniform corrosion process, specifically the critical thickness of the corrosion film. The parameter $K_{U}$ is tied to the kinetics of this process, while $L_{e}$ represents the characteristic finite element length. Additionally, $\lambda_{e}$ was introduced as an element-specific dimensionless pitting parameter. For every element on the initially exposed surface, a distinct random value for $\lambda_{e}$ was assigned using a standard random number generator based on the Weibull distribution.

**Figure 3:**
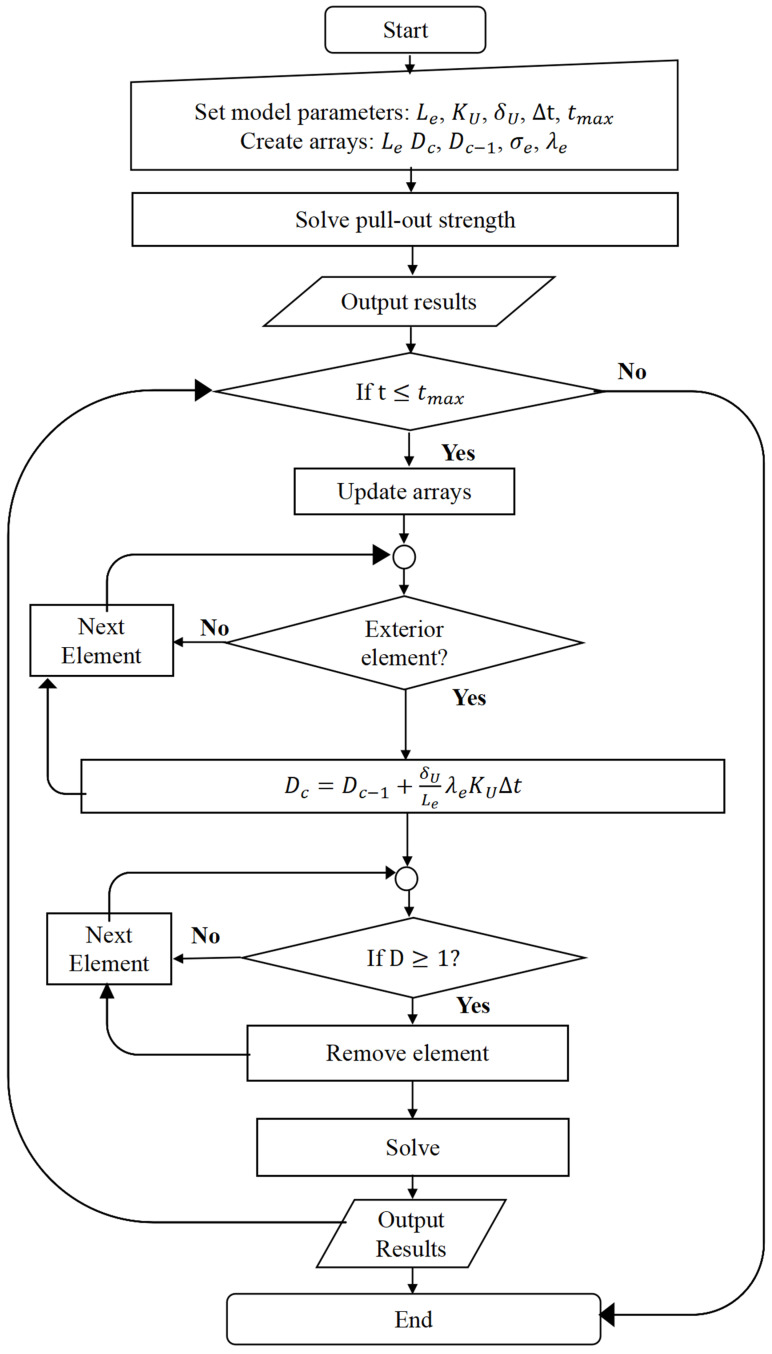
Flowchart of the corrosion algorithm.

The corrosion model was integrated into a finite element framework utilizing the Abaqus/Explicit solver and a user-defined subroutine (VUSDFLD), following the numerical algorithm illustrated in Figure 3. Initially, parameters were defined, and necessary arrays were initialized. The Abaqus/Explicit FE solver then evaluated the pull-out strength, and the resulting stress/strain data were transferred to the VUSDFLD subroutine. For each surface exterior element, damage was computed using Equation (2), which was applied in a discrete form to update the damage field at each time increment Δt:

(3) $$D_{c}=D_{c-1}+\frac{\delta_{U}}{L_{e}}\lambda_{e}K_{U}\Delta t$$

where $D_{c}$ from the previous iteration $D_{c-1}$ was utilized, with ∆t representing the time increment for calculations. This process was iteratively applied for every subsequent time step. As material damage progressively accumulated and $D_{c}$ attained a value of 1, the corresponding element was eliminated from the finite element mesh, and the corrosion surface was dynamically updated.

A uniaxial tensile test was conducted to calibrate the mechanical parameters of the material. Specimens with a circular cross-section of 5 mm in diameter and a gauge length of 10 mm were used for this test to ensure accurate calibration of the numerical model. A quasi-static axial loading was simulated in Abaqus/Standard by applying a displacement boundary condition to both ends of the sample, while all other boundaries were treated as free. The calibration process was completed by achieving the best fit to the experimental data.

Following model calibration, the screw pull-out from the bone was simulated. The bone was modeled as a rectangular block [32] with a cross-section of 20 mm×20 mm and a length of 30 mm, as shown in Figure 2b. The threaded hole was positioned at the center of the block, with its axis aligned along the length. The thread hole profile was designed to match the screw’s geometry. Figure 2c illustrates the loading condition where the screw was pulled out from the bone. The screw–bone interaction was simulated using the general contact algorithm in Abaqus, which enabled automatic redefinition of contact surfaces following element removal [33], allowing continuous modeling of the screw–bone interaction as corrosion progressed.

For tangential contact, the coefficient of friction was set to 0.2, based on Liu et al. [34]. Normal contact was defined as default “hard” contact. The vertebral trabecular bone was assumed to be homogeneous, isotropic, and perfectly plastic after yielding, with its yield strength and elastic properties derived from the published literature [35,36]. The evolution of the Mg damage factor is described by Equation (2), and the material properties of Mg-1Ca and bone are listed in Table 2.

A uniaxial displacement load was applied to the screw for the pull-out simulation, while the opposite side of the bone was fixed. Both the screw and the bone were meshed using C3D8R elements in Abaqus, which are eight-node reduced integration linear brick elements [37]. The optimal mesh size was determined to ensure computational convergence. To investigate mesh dependency, a series of computational trials were conducted. In one set of trials, the screw’s average mesh size was varied from 1.5 mm to 0.15 mm while maintaining the bone’s mesh size at 0.6 mm. In another set, the bone’s mesh size was varied from 2.0 mm to 0.4 mm, with the screw’s mesh size kept at 0.4 mm.

### 2.3. Objective Function

The objective of this optimization study was to maximize the long-term fixation performance of a candidate screw design under a corrosive environment. Pull-out strength is the most effective indicator of fixation performance when corrosion has progressed to a certain degree. Therefore, the objective function is defined in terms of pull-out strength. Clinically, mechanical performance is evaluated at key time points. In this study, pull-out strength and other mechanical properties were measured at 10% corrosion occurrence, which corresponds to approximately 6 weeks post-surgery according to Cheng et al. [38]. Further investigations will be required for other key time points.

Since the Sequential Quadratic Programming (SQP) algorithm searches for a minimum solution, the objective function was set as the negative pull-out strength. Due to the highly nonlinear nature of screw pull-out dynamics, deriving an analytical relationship between geometric parameters and pull-out strength is impractical. Moreover, obtaining pull-out strength through simulations for each iteration would be computationally expensive and time-consuming. To improve efficiency without compromising accuracy, this study employed a surrogate model constructed from a minimal set of sample points [39].

A surrogate model is a pre-selected approximation framework that captures the underlying relationship between input parameters and output responses. Once selected, the form of the model remains fixed, while its parameters are adjusted to achieve the best approximation. In this study, two surrogate models—Response Surface Methodology (RSM) and Kriging—were compared to determine the most effective approach.

The RSM was formulated as a full second-order polynomial with five design parameters, as expressed in Equation (4). At least 21 sample points are required to determine the polynomial coefficients through least squares analysis.

(4) $$y\left(x1,x2,\ldots ,x5\right)=a_{0}+\sum_{i=1}^{5}a_{i}x_{i}+\sum_{i=1,j=1,j\geq i}^{5}a_{ij}x_{i}x_{j}$$

The Kriging model is an interpolation technique based on Gaussian Processes, capable of providing both predicted values and estimates of prediction variance [40]. Initially rooted in geostatistics and spatial analysis, the Kriging method was designed to generate detailed maps of subsurface geological formations using data from a network of irregularly distributed borehole locations [41].

Constructing a Kriging model typically involves three key steps: selecting a trend function, choosing a correlation function, and estimating the correlation parameters.

(5) $$\hat{f}\left(x\right)=g{\left(x\right)}^{Τ}\beta +\epsilon \left(x\right)$$

In Equation (5), a Kriging emulator $\hat{f}\left(x\right)$ consists of a trend function (frequently a least square fit to the data $g{\left(x\right)}^{Τ}\beta$) plus a Gaussian process error model $\epsilon \left(x\right)$ that is used to correct the trend function. This reflects an approximate distribution for the unknown true surface $f\left(x\right)$. The error model $\epsilon \left(x\right)$ adjusts the trend function to ensure the emulator interpolates and exhibits zero uncertainty at the data points used in its construction. For further details on the Kriging model, refer to the study by Simpson et al. [42].

### 2.4. SQP Strategy

In this study, the SQP algorithm was employed to optimize surrogate models and obtain the optimal solution. SQP is a widely used general-purpose method for solving smooth nonlinear optimization problems, capable of evaluating function and gradient values with high precision [43].

Specifically, this study adopted an SQP algorithm with successive error restoration (NLPQLP) for both surrogate models. For more details on NLPQLP, refer to [44]. The surrogate models, combined with the NLPQLP algorithm, were implemented using Isight software (2019), which provides built-in support for these models and the optimization algorithm.

### 2.5. Optimization Flowchart

**Figure 4:**
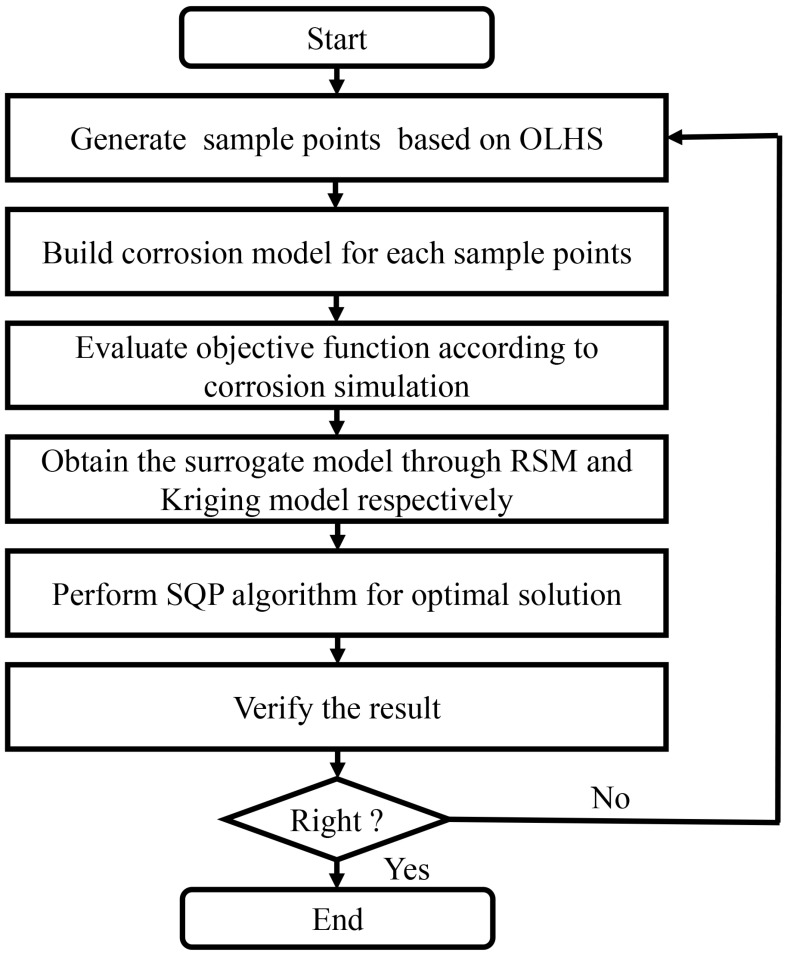
Flowchart of the optimization process.

In summary, the optimization process consisted of six steps, as illustrated in Figure 4.

(1) Sampling Initial Parameter Points: Initial parameter points were randomly selected using the Optimal Latin Hypercube Sampling (OLHS) technique [39,45].
(2) Generating Corrosion Models: Corrosion models corresponding to each sample point were generated using Abaqus/CAE (6.14) pre-processor and Python (2.73) scripting. The main function of the Python scripting is to traverse the elements in the model, mark the surface elements, and generate random numbers following a Weibull distribution for them. It then writes the element information, adjacent element information, and the random numbers into an Abaqus initial conditions file to set the initial conditions for the entire model.
(3) Evaluating the Objective Function: The objective function was assessed by running numerical simulations.
(4) Building Surrogate Models: Response Surface Methodology (RSM) and Kriging models were used to construct surrogate models that approximate the relationship between the objective function and geometric parameters.
(5) Optimization Using SQP Algorithm: The Sequential Quadratic Programming (SQP) algorithm was applied to explore the design space and determine the optimal solution.
(6) Verifying the Optimal Solution: The objective function was re-evaluated using the corrosion model built from the optimal solution. If the new objective function value was strictly lower than those of other sample points, the optimization process was complete. Otherwise, the process looped back to Step 1 and repeated.

## 3. Results

### 3.1. Calibration Results

**Figure 5:**
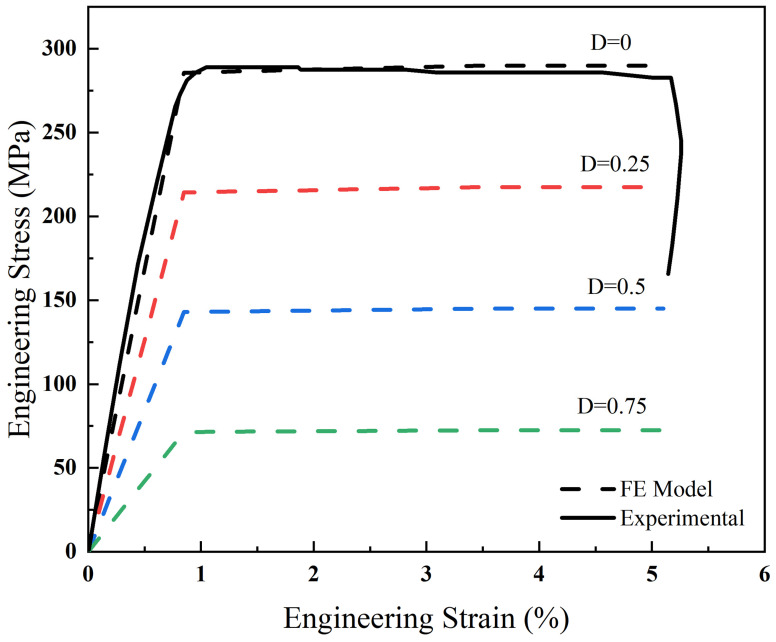
Representative engineering stress–strain curves for a uniaxial tensile test based on the results of experiments and simulations.

Figure 5 illustrates the stress–strain behavior of Mg-1Ca alloy, with solid lines derived from experimental data. The mechanical parameters for the numerical models, represented by black dotted lines, were optimized for material fit and are detailed in Table 2. Corrosion-induced degradation of mechanical integrity was modeled by adjusting material parameters using varying values of the damage field D, simulated in increments of 0.25 from 0 to 1. Assuming a direct correlation between load-bearing capacity reduction and D, stress–strain curves were generated by proportionally reducing the Young’s modulus of undamaged materials by D. As depicted in Figure 5, stress levels declined with increasing D at constant strain.

### 3.2. Mesh Sensitivity Analysis

**Figure 6:**
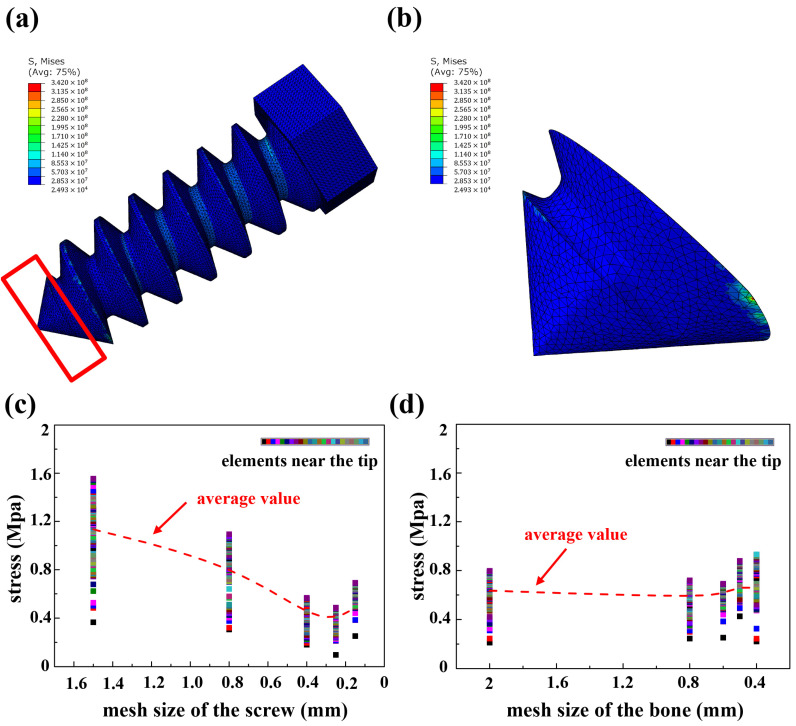
(**a**) Stress distribution on the screw of the first sample in Table 1. (**b**) Enlarge view of the part outlined in red in (**a**). The mesh sizes of the bone and screw were 0.4 mm and 0.25 mm, respectively. (**c**) Stress distribution of elements near the tip versus the mesh size of the screw (mesh size of the bone = 0.6 mm). (**d**) Stress distribution of elements near the tip versus the mesh size of the bone (mesh size of the screw = 0.4 mm).

It is essential to investigate the average mesh size that ensures convergence while minimizing computational time. The first group in Table 1 was used to explore this issue, and a series of computational trials, as described in the methodology section, were performed. Since the objective function is linked to stress distribution, and this study focused primarily on screws rather than bones, only the stress distribution on the screws was considered. The results are shown in Figure 6a, where a nearly uniform stress distribution with minor perturbations near the thread was observed. Given this characteristic of the stress distribution, it was unnecessary to examine the stress distribution of all elements, as it would have consumed excessive computational resources. Instead, a subset of elements near the screw tip, shown in Figure 6b, was selected to calculate and average the stress distribution. The proportion of different stress magnitudes in this subset was similar to the overall distribution.

Next, the mesh size of the screw was determined by keeping the bone mesh size constant (=0.6 mm). Figure 6c presents the stress distribution on the elements near the screw tip as a function of the screw mesh size. The data were obtained from the simulation when 10% of the screw had corroded. The results indicate that the stress distribution converged when the mesh size was smaller than 0.4 mm. Therefore, an average mesh size of 0.25 mm was selected for the screw in this study.

The bone mesh size was then determined by keeping the screw mesh size constant (=0.15 mm). The stress distribution versus the mesh size of the bone is shown in Figure 6d. Again, the data were collected when 10% of the screw had corroded. The results reveal that the stress distribution was relatively insensitive to the bone mesh size within the tested range, indicating that convergence had already been achieved. Thus, a mesh size of 0.4 mm was chosen for the bone. This further confirms that the earlier choice of 0.6 mm for the bone and 0.15 mm for the screw mesh sizes was appropriate.

### 3.3. Optimal Solutions

**Figure 7:**
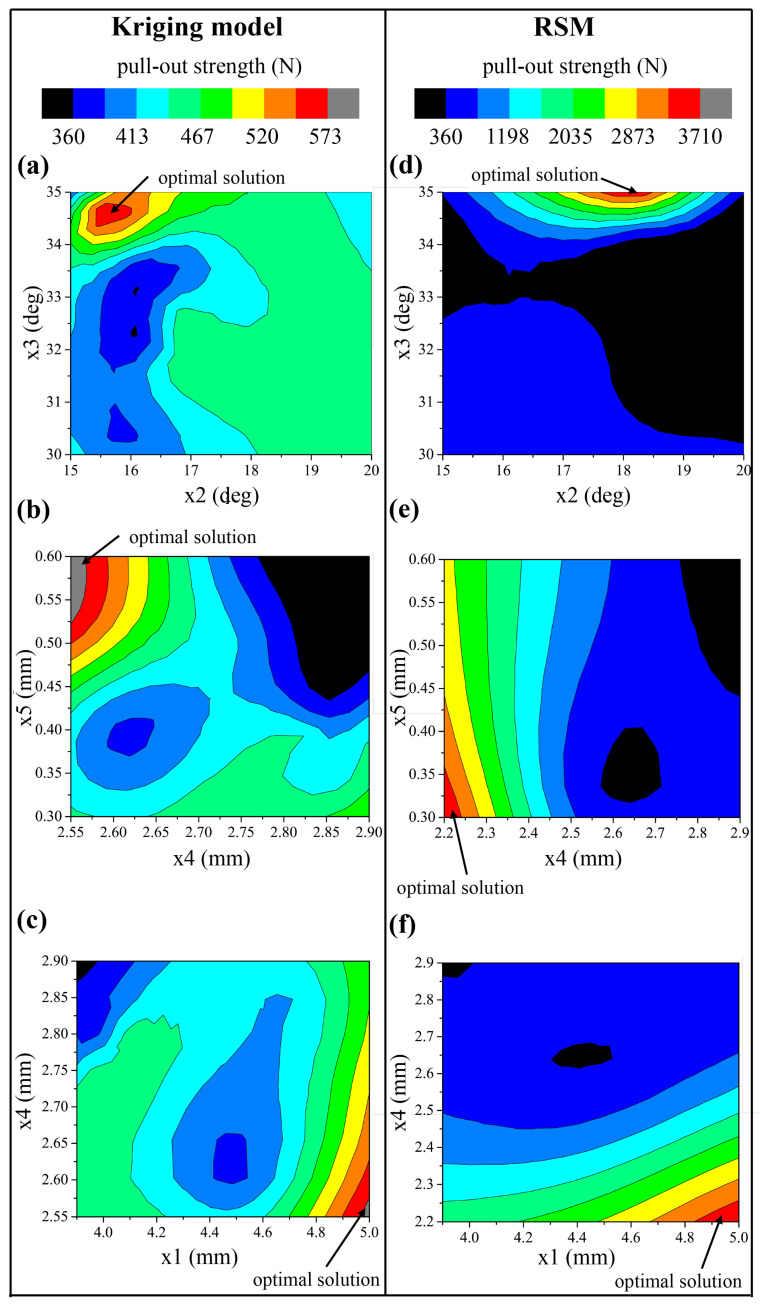
The projections of the five-dimensional response surface of the optimization results for the Kriging model in (**a**) the $x_{2}-x_{3}$ plane, (**b**) the $x_{4}-x_{5}$ plane, and the (**c**) $x_{1}-x_{4}$ plane; and for RSM (**d**) the $x_{2}-x_{3}$ plane, (**e**) the $x_{4}-x_{5}$ plane, and the (**f**) $x_{1}-x_{4}$ plane.

The results of a five-dimensional optimization should be a five-dimensional response surface. However, it cannot be exhibited in this three-dimensional world directly. Therefore, the projections of the five-dimensional surface in three planes shown in Figure 7 were taken to research the rules. These three projection planes can cover all the five dimensions that are cared about.

For the Kriging model, Figure 7a–c exhibit the distribution of pull-out strength with respect to $x_{2}-x_{3}$, $x_{4}-x_{5}$, and $x_{1}-x_{4}$, respectively. It can be found in Figure 7a that the maximum pull-out strength occurred when $x_{2}$ was located between 15.5° and 16.0°; meanwhile, $x_{3}$ located near 34.5°. Near the maximum value, the pull-out strength gradually diminished outward from the center, forming a layered distribution similar to concentric circles. However, this rule was invalid far away from the maximum value, especially at the bottom left of the figure where the minimum value occurred. The maximum pull-out strength lay in the top left corner of the Figure 7b, and pull-out strength decreased layer by layer outward, with the maximum value as center. The decreasing trend extended towards two local minima values in the top right and bottom left directions in the figure. However, it is noteworthy that, in the bottom left and bottom right corners of the figure, the pull-out strength showed a slight increase. The maximum pull-out strength lay in the bottom right corner of the Figure 7c, and pull-out strength decreased layer by layer outward, with the maximum value as center. The decreasing trend extended towards two local minima values in the top left and middle-lower directions in the figure. In addition, it can be found that the maximum pull-out strength corresponded to an $x_{4}$ value around 2.55 mm, which is consistent with Figure 7b.

For the second-order RSM, Figure 7d–f exhibit the distribution of pull-out strength with respect to $x_{2}-x_{3}$, $x_{4}-x_{5}$, and $x_{1}-x_{4}$, respectively. It can be found in Figure 7d that the maximum pull-out strength occurred when $x_{2}$ was around 18° and $x_{3}$ was around 35°. Pull-out strength decreased layer by layer outward, with the maximum value as center. However, it is noteworthy that, in the bottom of the figure, the pull-out strength showed a slight increase. The maximum value was located at the bottom left corner in Figure 7e, and pull-out strength decreased layer by layer outward, with the maximum value as center. The decreasing trend extended towards two local minima values in the top right and middle-lower directions in the figure. The maximum pull-out strength lay in bottom right corner in Figure 7f, and pull-out strength decreased layer by layer outward, with the maximum value as center. The decreasing trend extended towards two local minima values in the top left and middle directions in the figure. In addition, it can be found that the maximum pull-out strength corresponded to an $x_{4}$ value around 2.2 mm, which is consistent with Figure 7e. The optimal solutions of the two models are listed in Table 3.

## 4. Discussion

### 4.1. The Relationship Between Pull-Out Strength and Design Parameters

For the Kriging model, pull-out strength was not monotonous about both $x_{2}$ and $x_{3}$ in Figure 7a, and the law was complicated. Pull-out strength increased with $x_{4}$ getting smaller and $x_{5}$ getting bigger in Figure 7b. Pull-out strength increasing with $x_{4}$ getting smaller was proved again in Figure 7c and correlated positively with $x_{1}$.

For the second-order RSM, the two sides of the optimal $x_{2}$ were symmetric in Figure 7d. Different from $x_{2}$, the pull-out strength monotonically increased with respect to $x_{3}$. In Figure 7e, pull-out strength was negative correlated with both $x_{4}$ and $x_{5}$. Pull-out strength being negatively correlated with $x_{4}$ was proved again in Figure 7f, and it increased with $x_{1}$ becoming bigger.

It is obvious that the contour plot of RSM had clearer rules than the Kriging model in terms of monotony, and the layers of RSM were more smoothing, with fewer irregular protuberances. $x_{1}$ and $x_{4}$ had the same monotony, while $x_{5}$ had opposite monotony for the two surrogate models. Both models indicate that pull-out strength was not monotone with respect to $x_{2}$, but the optimal values given by them were different. It is interesting that the pull-out strength given by RSM increased monotonically with respect to $x_{3}$, while the pull-out strength obtained from Kriging model was not monotone, with the optimal value being about 34.5°. The Kriging model considers random processes, so it cannot be so smooth as RSM. Simply due to considering random processes, the Kriging model greatly improves the accuracy of prediction.

### 4.2. Verification

The structural optimization process generated two solutions independently using the SQP algorithm for each surrogate model. However, these solutions needed to be validated through finite element analysis (FEA), from which the sample points were originally derived. To verify the results, two separate FEA simulations were conducted based on the optimized parameters while maintaining the same corrosion model as the sample points.

**Figure 8:**
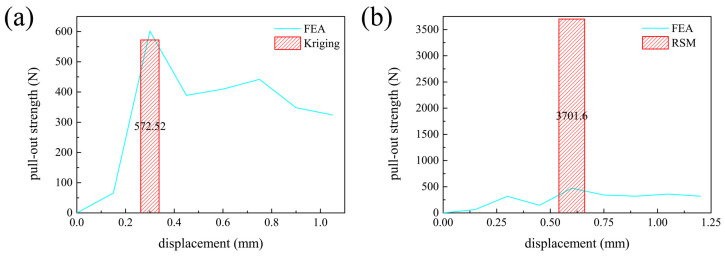
Pull-out strength versus displacement, as determined by (**a**) Kriging model and (**b**) RSM at the time when 10% of the screw had corroded.

As shown in Figure 8a, the pull-out strength obtained from FEA closely matched the prediction from the Kriging model, with an error of less than 5%. Additionally, it was higher than any value in the sample set, confirming the validity of the Kriging-based optimization. In contrast, the FEA results for the second-order response surface model (RSM), depicted in Figure 8b, showed a significantly lower pull-out strength than the RSM-predicted value. Moreover, it was not the highest value within the sample set, suggesting a discrepancy between the surrogate model and the actual physical behavior.

This discrepancy highlights the importance of selecting an appropriate surrogate model in optimization problems. Since the surrogate model serves as an approximation of an unknown phenomenon, an inaccurate model will fail to produce reliable optimization results, regardless of the algorithm used. The findings indicate that, while the Kriging model accurately captured the relationship between the design parameters and the objective function, the second-order RSM did not adequately represent these complex interactions.

Therefore, the optimization results obtained using the Kriging model were deemed reliable and acceptable for this study.

### 4.3. Rationality

**Figure 9:**
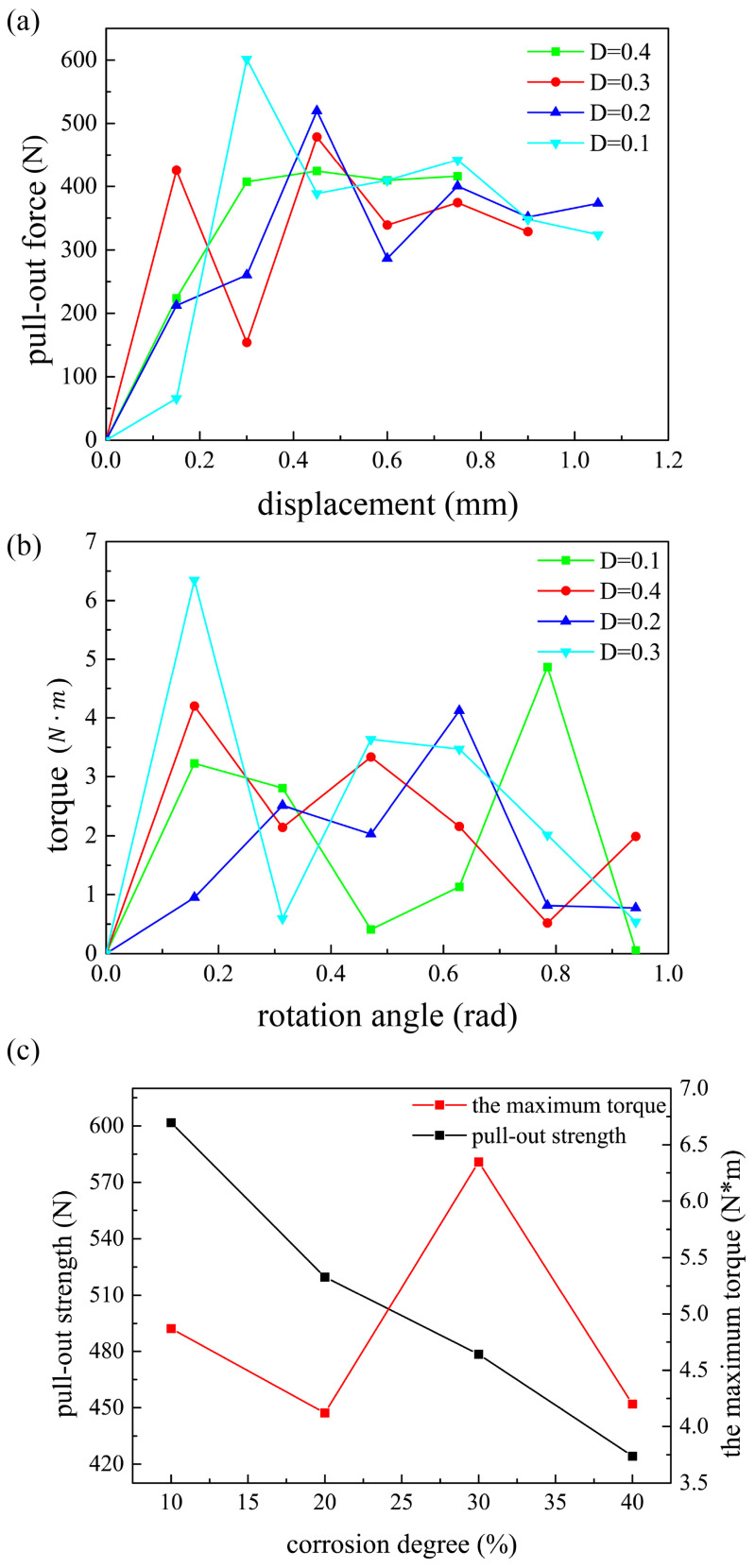
(**a**) The relationship between pull-out strength and displacement under different corrosion degrees. (**b**) The relationship between torque and rotation angle under different corrosion degrees. (**c**) Pull-out strength and the maximum torque evolve with corrosion progression.

In view of the optimization result obtained from Kriging model, we performed further analyses. First, the changes in pull-out strength when corrosion progressed to different degrees were studied. Figure 9a shows the relationship between pull-out force and displacement when the screws were corroded to 10%, 20%, 30%, or 40%. The pull-out strengths are shown in Figure 9c. It can be found that, as the degree of screw corrosion increased, its pull-out strength gradually declined. This trend is consistent with clinical observations. Once the degree of corrosion is too large, the geometric structure will be severely deformed, and the mechanical boundary conditions will also change. Therefore, the model only studied the corrosion degree of 40% at most, and more serious corrosion conditions were not considered.

In addition to the pull-out strength, we also studied the torque of the screw. All the analysis steps were the same as those performed for pull-out strength, except the displacement load in Figure 2c was replaced by rotation angle. Figure 9b shows the relationship between torque and rotation angle when the screws were corroded to 10%, 20%, 30%, or 40%. The maximum torques are shown in Figure 9c. Unlike the pull-out case, the torque did not gradually decrease with the corrosion progression, and there was no obvious rule to follow. This also proves that the selection of pull-out strength as the optimization target instead of torque was the correct decision in this work.

**Figure 10:**
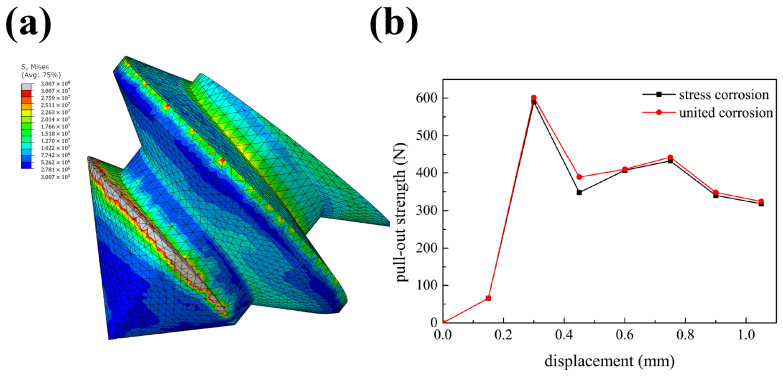
(**a**) Stress distribution near the screw thread. (**b**) The relationship between pull-out strength and displacement when the screw was corroded to 10% for the stress corrosion model and the uniform corrosion model.

According to the stress distribution results shown in Figure 10a, considering that the stress near the screw thread is large, this study further used the stress corrosion model to replace the uniform corrosion model to reanalyze the pull-out strength of the optimized result. The two results are compared in Figure 10b. It can be found that the introduction of stress corrosion made the pull-out curve slightly lower than that under the uniform corrosion case, but its influence on the pull-out strength was very limited. This result indicates two points. First, the uniform corrosion model used in this work as a representative for the optimization process instead of a more complex corrosion model had little effect on the pull-out strength. The optimization result in this paper is credible to a certain extent. Second, although the effect of stress corrosion was not obvious, it was still harmful to screw fixation. The thread where the stress distribution is large will corrode more seriously, and might lead to premature failure. This can explain the phenomena in the experiments performed by Huang et al. [21]. It further implies that the thread where the stress distribution is large should be thickened appropriately so that the screw can have a longer fixing time.

### 4.4. Limitations

Although this paper gives optimal geometric parameters of a Mg-1Ca interference screw, it still has some shortcomings due to methodological limitations. First, a pitting corrosion model was adopted when FEA was performed, but corrosion is more complicated in a real physiological environment. A simplified pitting corrosion model cannot capture all the features, which may have introduced error.

In addition, there are many surrogate models and optimization algorithms that could have been used to optimize this problem. SQP, RSM, and the Kriging model are frequently used ones. They were adopted in this work, but whether other surrogate models and optimization algorithms may yield a better solution still remains to be explored.

Furthermore, all possibilities should be exhausted to verify the optimal solution theoretically. However, no algorithm can search infinite points in the design space. Thus, the initial sample points can also influence the optimization result, in addition to the optimization algorithms and surrogate model. Different result may be attained even with the same surrogate model and optimization algorithms based on disparate initial sample points. This analysis was beyond the scope of the present study and requires further investigation.

This study aimed to optimize parameters based on existing screw structure instead of creating a new structure. Designing a new structure is promising, but was beyond the scope of this study.

The optimization result given by this paper is based on a Kriging surrogate model and SQP optimization algorithm. It was verified through FEA, with error less than 5%, and better than all the points in the sample set. Although it cannot be certain whether there is another solution in the design space better than this solution, it is still an acceptable solution and will perform better than the existing design.

## 5. Conclusions

This study optimized the structure of Mg-1Ca interference screws, considering the degradability of magnesium, using the CDM (Continuum Damage Mechanics) model. Both Response Surface Methodology (RSM) and Kriging surrogate models were employed, with optimization performed using the Sequential Quadratic Programming (SQP) algorithm.

The Kriging model produced an acceptable solution, while the RSM model was found to be inappropriate, as validated by finite element analysis (FEA).

The final result includes the optimized design parameters: outer diameter 7 mm, length 25 mm, proximal root radius 0.2 mm, distal root radius 0.4 mm, inner diameter 4.96 mm, proximal half angle 15.52°, distal half angle 34.57°, pitch 2.55 mm, and thread width 0.52 mm. The pull-out strength (601.69 N) of this optimized design, as calibrated by FEA, demonstrates a significant improvement over the baseline configuration. This paper not only summarizes the key findings—such as the critical role of geometric parameters in enhancing mechanical performance—but also proposes specific future research directions. For instance, extending this framework to other alloys could provide valuable insights into the generalizability of the optimization approach, paving the way for broader applications in materials science and engineering.

## Figures and Tables

**Table 1:** Geometric parameters of interference screws.

Group	$x_{1}(mm)$	$x_{2}({}^{\circ})$	$x_{3}({}^{\circ})$	$x_{4}(mm)$	$x_{5}(mm)$
1	4.5	15.0	30.0	2.75	0.40
2	3.9	16.4	32.3	2.85	0.42
3	5.0	15.0	30.0	2.75	0.40
4	4.0	16.1	33.5	2.88	0.45
5	4.5	15.0	30.0	2.75	0.50
6	4.3	16.6	32.5	2.80	0.39
7	4.5	15.0	30.0	2.60	0.40
8	4.2	18.7	30.1	2.76	0.30
9	4.5	15.0	35.0	2.75	0.40
10	4.3	16.6	31.9	2.55	0.32
11	4.5	20.0	35.0	2.75	0.40
12	4.0	15.8	30.4	2.80	0.45
13	4.5	20.0	30.0	2.75	0.40
14	4.6	16.8	33.8	2.64	0.43
15	5.0	20.0	30.0	2.90	0.30
16	4.0	15.0	30.0	2.75	0.40
17	4.2	17.4	32.5	2.83	0.39
18	4.5	15.0	30.0	2.75	0.30
19	4.7	15.2	32.5	2.84	0.36
20	4.5	15.0	30.0	2.75	0.60
21	4.1	18.2	34.2	2.79	0.31
22	4.5	15.0	30.0	2.90	0.40

**Table 2:** The material properties of Mg-1Ca and bone.

**Mg-1Ca**	**Young Modulus (MPa)**	**Poisson’s Ratio**	**Density** **(kg/m^3^)**	**Yield Stress (MPa)**	**Plastic Strain (%)**
	43,480	0.3	1738	277	0
				290	0.0106
**Bone**	**Young Modulus (MPa)**	**Poisson’s Ratio**	**Density** **(kg/m^3^)**	**Yield Stress (MPa)**	**Plastic Strain (%)**
	100	0.2	1200	2	0

**Table 3:** Optimization results of the Kriging model and RSM.

	$x_{1}\;(mm)$	$x_{2}({}^{\circ})$	$x_{3}\;({}^{\circ})$	$x_{4}\;(mm)$	$x_{5}\;(mm)$	Pull-Out Strength (N)
Kriging	5.0	15.5	34.6	2.55	0.52	572.5
RSM	5.0	18.2	35.0	2.20	0.30	3701.6

## Data Availability

Data is contained within the article.
